# Intestinal Stem Cell-on-Chip to Study Human Host-Microbiota Interaction

**DOI:** 10.3389/fimmu.2021.798552

**Published:** 2021-12-06

**Authors:** Fatina Siwczak, Elise Loffet, Mathilda Kaminska, Hristina Koceva, Maxime M. Mahe, Alexander S. Mosig

**Affiliations:** ^1^ Center for Sepsis Control and Care & Institute of Biochemistry II, University Hospital Jena, Jena, Germany; ^2^ Université de Nantes, Inserm, TENS, The Enteric Nervous System in Gut and Brain Diseases, IMAD, Nantes, France; ^3^ Department of Pediatric General and Thoracic Surgery, Cincinnati Children’s Hospital Medical Center, Cincinnati, OH, United States; ^4^ Department of Pediatrics, University of Cincinnati, Cincinnati, OH, United States

**Keywords:** stem cell, gut-on-chip, microbiota, intestine, host-microbiota interaction, *in vitro* model

## Abstract

The gut is a tubular organ responsible for nutrient absorption and harbors our intestinal microbiome. This organ is composed of a multitude of specialized cell types arranged in complex barrier-forming crypts and villi covered by a mucosal layer controlling nutrient passage and protecting from invading pathogens. The development and self-renewal of the intestinal epithelium are guided by niche signals controlling the differentiation of specific cell types along the crypt-villus axis in the epithelium. The emergence of microphysiological systems, or organ-on-chips, has paved the way to study the intestinal epithelium within a dynamic and controlled environment. In this review, we describe the use of organ-on-chip technology to control and guide these differentiation processes *in vitro*. We further discuss current applications and forthcoming strategies to investigate the mechanical processes of intestinal stem cell differentiation, tissue formation, and the interaction of the intestine with the microbiota in the context of gastrointestinal diseases.

## Introduction

The gastrointestinal tract is a central organ system that enables the ingestion, digestion, absorption, and the utilization of processed nutrients to fuel the overall body. Within this system, several organs are responsible for digestion and the uptake of nutrients, including the stomach, the small intestine, and the large intestine. The gut forms a tubular structure whose central lumen is enclosed by a protective mucosa composed of a monostratified epithelium forming invaginations (or crypts) and finger-like protrusions (or villi). The epithelium allows nutrient uptake and acts as the first line of protection against invading pathogens (1). A crypt-villus structure is seen in the small intestine, while only crypts are present within the colon. Both the initial development and continuous self-renewal of the intestinal epithelium are guided by niche signals in addition to cell-autonomous processes that initiate the outgrowth and differentiation of intestinal stem cells (ISCs), driving proliferation and differentiation along the crypt-to-villus axis in the small intestine. Transit-amplifying cells derived from ISC further separate into lineages showing absorptive or secretory characteristics that terminally differentiate into specific cell types within the epithelium (2). Enterocytes are the predominant cell type in the epithelium and involved in the absorption and transport of small molecules from the intestinal lumen into the bloodstream. Enterochromaffin cells, a enteroendocrine cell type, are specialized cells of the epithelial tissue that secrete hormones and modulate neural activity by releasing neurotransmitters (3). Microfold cells (M cells) are responsible for the transport of luminal antigens to the lymphoid follicles, thereby initiating an adapted immune response to the microbiota (4). The composition of the microbiota at the epithelium can be modulated by Paneth cells through secretion of antimicrobial peptides. In addition, this cell type contributes to the maintenance of the ISC niche (5). Tuft cells and cup cells belong to the rarer cell types of the intestinal epithelium. For Tuft cells, a contribution to neural signaling has been proposed (6) whereas the function of cup cells still awaits clarification. This complex epithelium composed of several cell types is surrounded by smooth muscle cell layers, the enteric nervous system, and connective tissue that contains arteries and lymphatics and is abundant in fibroblasts and mast cells (7).

The last few decades in biomedical research have led to a number of robust experimental strategies and techniques to study intestinal physiology. Yet, *in vitro* studies aiming to model the gut mucosa have mostly been limited to static monocultures and co-cultures. In contrast to these older models, recent developments that use microphysiological systems allow scientists to probe both the intestinal epithelium and its environment in a dynamic and physiologically relevant manner (8). In this review, we will emphasize the transitions from immortalized cell lines to stem cells and describe their use in combination with gut-on-chip models. These “on-chip” models are defined as three-dimensional (3D) cell cultures arranged as multiple cell layers actively perfused by microfluidics for medium exchange to enable an improved tissue formation with increased lifespan. We will highlight selected applications of these gut-on-chip models to understand gastrointestinal diseases and forthcoming strategies to investigate human gut physiology.

## Cell Sources for Gut-on-Chip Models

The recapitulation of whole embryonic development can be challenging due to a lack of knowledge of all the required biochemical cues driving the differentiation and morphogenic processes. One of the most novel *in vitro* approaches in intestinal mucosa modeling is the use of microfluidically perfused organ-on-chip seeded with cell lines, primary cells, or stem cells (9–14). A clear benefit of these models is that they allow for better control of the microenvironment. One example includes the addition of flow on Caco-2 cells to differentiate them and express the typical cell markers of essential cell types of the small intestine (Goblet- and Paneth-, enterochromaffin-, and enterocytes) (15). Further, flow conditions stimulated an increased expression of mucin-2 with the development of a thickened mucus layer covering the epithelial tissue, indicating the functional relevance of flow conditions for improved goblet-like cell functionality (9, 16). Perfused organ-on-chip further facilitates physiological cellular crosstalk by aiding in the outgrowth of organotypic microstructures such as villi and crypts (9, 12). In addition, an epithelial cell layer has been combined in a number of studies with endothelial cells and immune cells using various tissue engineering approaches (9, 12, 17). These studies highlight the potential of organ-on-chip as a tool to manipulate the microenvironment by facilitating the long-term growth and co-differentiation of different cell types to replicate some typical microanatomical features of the human intestine.

## Control and Guidance of Intestinal Cell Development on-Chip

Immortalized cell lines have been used extensively to study the intestine. For example, the colorectal adenocarcinoma cell line Caco-2 had been originally used to study the intestinal epithelial barrier as these cells differentiate spontaneously into a monolayer of enterocytes when reaching confluence (18). However, given the cancerous background of the Caco-2 cell line data generated with these models, the data should be interpreted with caution as this cell line has several limitations in its differentiation potential to individual intestinal cell types compared to adult stem cells or induced pluripotent stem cells (19). Further, immortalized cell lines such as Caco-2 cannot reflect a patient-specific genetic background to enable a personalized approach to study individual mechanisms of disease-related conditions.

To overcome these limitations, the use of human pluripotent stem cells (hiPSCs) or adult stem cells has been explored as a promising alternative to established cell lines. Human adult ISCs maintain an organ-specific imprint and epithelial maturity and possess a patient-specific background, thus enabling personalized studies (9, 14). Biopsy-derived cells and intestinal organoid models have emerged as powerful tools to mimic tissue complexity and high cellular diversity *in vitro* (20, 21). ISCs are a valuable cell source to grow organoids with organotypic self-patterning, thereby recreating essential microanatomical features of the gut (22). However, the availability of primary tissue for the isolation of ISCs is limited. Hence, hiPSCs are an exciting alternative as they can generate intestinal cells through directed differentiation (23). The use of hiPSCs allows for the generation of multiple differentiated cell types from a patient-specific background with an unlimited supply of human stem cells stored in biobanks (24). Furthermore, hiPSCs from patients with a genetic background of interest can be obtained by reprogramming from a multitude of easily accessible primary cell sources, including urine, blood, and skin (25). Nevertheless, a significant drawback in using pluripotent stem cells can be found in difficulty producing tissues that reach full maturity.

**Figure 1:**
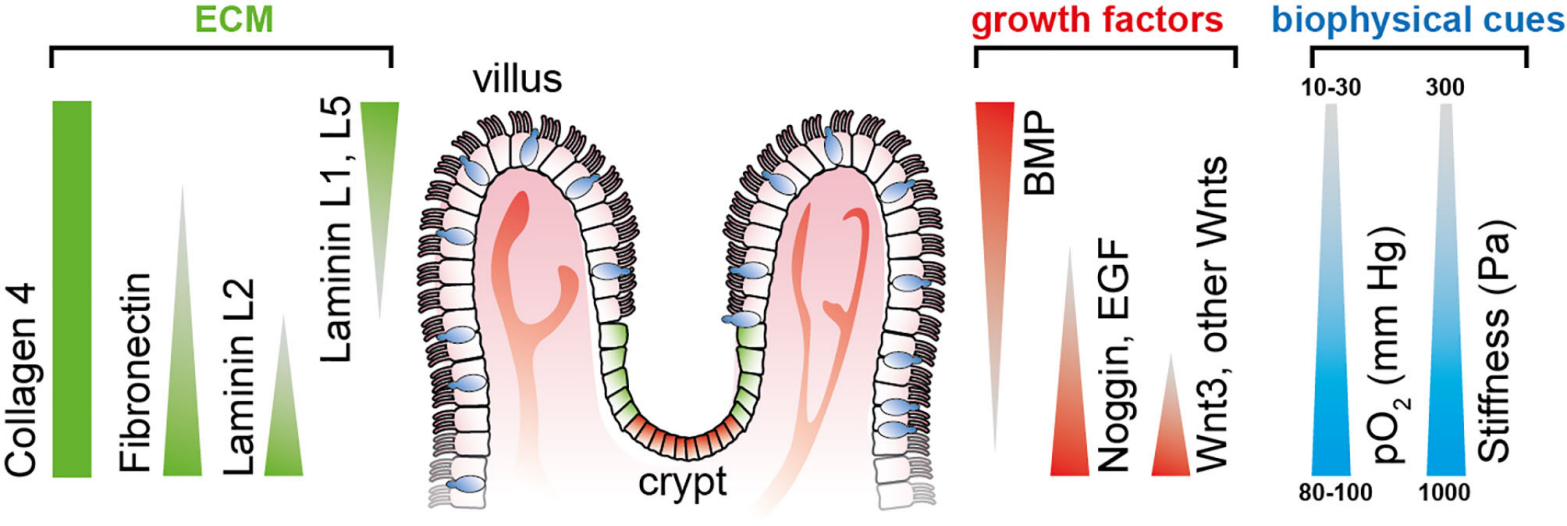
Defined gradients of biochemical and biophysical cues span along the crypt-villus axis in the small intestine, thereby determining cellular stemness and differentiation toward the tips of the villi. Triangles indicate the gradient direction of growth factors and ECM protein. Illustration adapted from (26).

ISC-derived organoids have been used to experimentally address and dissect the individual spatiotemporal effects of different biochemical gradients on the growth and differentiation of ISCs. However, given the spherical nature of organoids, the stable recreation of physiological conditions over a more extended period of culture has remained challenging (26). Microfluidically perfused organ-on-chip platforms mimic *in vivo-*like biochemical cues to guide stem cell differentiation with typical microanatomical features such as villi and crypts (**Figure 1**).

It has been long known that many aspects of cell fate are dependent on the geometry and mechanics of their microenvironment, including the induction of apoptosis (27), cellular differentiation (28, 29), and proliferation rate (30). The presence of a flow-through media stream has been demonstrated to be a requirement for forming 3D intestinal structures in cell lines and primary cell-based intestinal models by modulating the biochemical availability of growth factors. The flow-dependent basolateral removal of the Wnt antagonist Dickkopf-1 and the elevated expression of the Frizzled-9 receptor was required in the formation of epithelium villi-like protrusions (31). Our existing knowledge on the individual growth factors driving ISC differentiation provides an excellent basis to engineer more realistic organ models with better accessibility. In this review, we are focusing on the use of the following two principal types of stem cells to differentiate and form intestinal tissue: adult stem cells and induced pluripotent stem cells. Both cell sources possess unique advantages and disadvantages in recapitulating the intestinal tissue (**Table 1**).

**Table 1:** Pros and cons of the use of adult stem cells and induced pluripotent stem cells for the generation of intestinal tissue models.

Cell Source	Advantages	Disadvantages
Adult Stem Cells (ASCs)	- semi-autonomous multipotent differentiation capacity (32) - preservation of tissue- and region-specific characteristics	- could not be differentiated to non-epithelial tissue (endothelial cells, immune cells, etc.) - limitations for expansion to large scale - limited by the accessibility of tissue
Induced Pluripotent Stem Cells (iPSCs)	- differentiation guided by external factors - somatic cells for reprogramming are easily accessible - pluripotent and useable for generation of isogenic models with high cellular diversity - lower frequency of DNA mutations during *in vitro* culture compared to ASCs (33), preservation of genetic identity *in vitro* over a more extended period of time	- variations in differentiation efficacy with limitations in cellular maturity - heterogeneity in the generation of tissue-specific cell types

A striking example of the potential of defined growth factor gradients to guide stem cell growth to intestinal tissues has been given by Wang et al. in the Transwell system (34, 35). Arrays of artificial collagen-based crypts and villi structures were created by microfabrication and molding to mimic the topography of the intestine and the colon (34, 35). The scaffold has been used to generate gradients of Wnt-3A, R-spondin, noggin, and gamma-secretase inhibitor. These gradients enabled the establishment and maintenance of a crypt-villus axis composed of adult human ISCs that formed an epithelium containing proliferating progenitor cell compartments. Kasendra et al. recently presented a more complex model of the human duodenum that combines the advantages of organoids and organ-on-chip technologies (9, 36). In this model, the tissue forms a polarized cell architecture under flow conditions and shows improved intestinal barrier function and differentiation of specialized cell subpopulations. This duodenum-on-chip system could exert mechanical stimulation to organoid-derived epithelial cells, resulting in improved tissue architecture. The tissue perfusion significantly improved cell-cell junctions and microvilli density, further improving intestinal barrier function. Remarkably, it was demonstrated that organoid-derived cells in the duodenum chip have an increased similarity in their global gene expression profile to human adult duodenal tissue compared to conventional static organoid culture. Further, in the duodenum-on-chip system, organoid-derived cells show an improved metabolic capacity compared to Caco-2 cells due to an increased induction capability and expression of CYP3A4 (36). The ability to integrate and analyze patient-specific stem cells from different donors within the chip represents a decisive advantage over the use of immortalized cell lines such as Caco-2 or HT-29. Stem cells allow for the reflection of the host’s genetic background and possess the ability to self-organize into an *in vivo*-like tissue structure with improved functionality. Both aspects are significant assets for studies on drug uptake and drug-drug interaction and its metabolization to predict drug efficacy and safety. Recently, Nikolaev et al. used synthetic hydrogels to guide the growth of adult human ISCs with intrinsic self-organization properties to tubular structures forming crypt- and villus-like domains (37). Furthermore, conventional organoids and 2D cell layers derived from intestinal organoids possess a high regenerative potential when cultured on hydrogels, as demonstrated after stimulation with cytotoxic dextran sodium sulfate (DSS) to model colitis. The perfusion of the system was used to provide a continuous supply of nutrition and the removal of waste products and dead cells, allowing the formation of a tenfold higher cell mass and the emergence of rare cell types such as M cells (37).

Similar approaches have also been demonstrated using hiPSCs. Workman et al. used epithelial cells isolated from hiPSC-derived human intestinal organoids and seeded them in a microfluidic perfused gut-on-chip. In the 3D tissue model, the spontaneous differentiation into epithelial-specific types, such as Paneth cells, goblet cells, enterocytes, enteroendocrine cells, transit-amplifying cells, and Lgr5+ cells similar to *in vivo* cell populations was observed under flow conditions (38). The immune responsiveness of the intestinal tissue after INFγ stimulation was validated by the measurement of STAT1 phosphorylation after one hour and the upregulation of INFγ−related genes three days after stimulation. Further, combined treatment with INFγ and TNF resulted in increased permeability of the epithelial cell layer. In another study, the same chip platform was used to culture stem cell-derived epithelial cells isolated from human colon organoids (14). Likewise, the OrganoPlate system, a plate-based cell culture system that allows pumpless media movement *via* a rocking platform, was used for cultivating hiPSC on a gel matrix to form a monolayered epithelial tube (39). The cells expressed the Paneth cell marker Lysozyme, the enterocyte cell marker Villin-1, and the neuroendocrine cell marker Chromogranin A. However, the expression of these cell marker proteins was diffuse and distributed over the entire tissue. Another shortcoming of the model is the absence of organized 3D epithelial structures.

These gut-on-chip systems strikingly demonstrate the vast potential of organ-on-chip platforms to precisely control environmental growth conditions, to guide the differentiation of stem cells, and to form improved intrinsically self-organized tissue with superior properties compared to traditional organoid cultures. Hence, the applications of ISCs include the possibility to investigate intestinal developmental biology and barrier function but also studies related to bacterial colonization, drug discovery, regenerative medicine, and responses to infections in a personalized approach (40–43). The generation and integration of hiPSC-derived immune cells such as T cells, NK cells, and macrophages by use of differentiation protocols that became recently available will aid in the development of isogenic immune-competent intestinal models derived from a single hiPSC line to avoid potential allogenic reactions (44–46).

## Gut-on-Chip Models to Study Microbiome-Host Interaction

As a result of coevolution, niches of the human body are inhabited by a variety of commensal, mutualistic, and pathogenic microorganisms, including archaea, bacteria, fungi, phages, protists, and viruses (47–53). The gut provides a variety of niches serving as habitats for different ecological communities. On a macroscopic scale, physiological gradients determine the growth niche of microorganisms along the gut from the small intestine toward the rectum with an increase in the pH and the abundance of bile acids, whereas the availability of oxygen decreases. A change in the environmental conditions thereby determines microbial colonization with 10^4^ CFU/ml bacteria in the small intestine up to 10^8^ CFU/ml microorganisms in the terminal ileum, where elongated passage times and associated enrichment in nutrients provide ideal bacterial growth conditions (54, 55). With that, the number of facultative anaerobic and obligate anaerobic species increases from the small intestine to the colon (56, 57).

Toll-like receptors (TLR) belong to a class of pattern recognition receptors evolutionary highly conserved and centrally involved in mediating host-microbe interactions in epithelial cells (58). Recent studies provide evidence of a defined spatial organization of TLR expression along the gut axis, which seems to be determined already before birth in tissue-resident stem cells and is only partially dependent on the interaction with the commensal gut microbiota (59, 60). TLR dependent pathways have been shown to mediate not only antimicrobial functionality but also directly contribute to the regulation of epithelial cell differentiation, which is of particular importance for the intestine (61). LPS activation of TLR-4 enhances cell differentiation of goblet cell lineages in colonic organoids but inhibits Lgr5+ proliferation with induction of apoptosis in stem cells. Similar observations have been made in intestinal organoids (62). MyD88 is an essential adapter protein to TLR-4 and critically involved in mediating LPS dependent maintenance of mucus production. Consequently, MyD88-/- mice have impaired mucus production with increased bacterial adherence to epithelial cells and loss of barrier function (63). TLR activation has also been demonstrated to contribute to the polarization of IECs depending on the side of stimulation (64). A strict unique spatial distribution pattern of TLRs between the apical or basolateral side of IECs along the gut axis contributes to an adapted interaction with the microbiota as a requirement for the maintenance of barrier functionality (65, 66). The proof of a physiological TLR distribution pattern in IECs cultured in gut-on-chip models remains an open task, but likely represents an important requirement to faithfully recapitulate host-microbiota interaction *in vitro*. IECs derived from different gut sections cultured in gut-on-chip and precisely stimulated with TLR agonists in a precise spatiotemporal manner by microfluidic perfusion would offer an interesting approach for achieving a physiologically relevant TLR expression pattern *in vitro.* Recent work already demonstrated the feasibility of a similar approach to achieve intestinal tissue polarization in an immunocompetent gut-on-chip model. Monocyte derived macrophages and dendritic cells here instructed immune tolerance and the maintenance of barrier function depending on the site of LPS stimulation (12).

The reliable monitoring of the microenvironment and its change upon tissue formation or bacterial colonization is key to control and understand the stem cell development and differentiation as well as the interaction of the intestinal tissue with the microbiota. Luminescent-based sensors integrated into organ-on-chip systems have been used to quantify medium dissolved oxygen levels (67, 68), cellular glucose consumption (69), and the changes of the pH by the release of lactate by metabolic active tissues or microorganisms (70–72). To enable the growth of anaerobic bacteria in gut-on-chip, several platforms have been tailored to allow the stable formation of neighboring cell layers perfused with normoxic and hypoxic media streams. The characterization and quantification of microbiota-associated changes in the metabolic profile require reliable and continuous measurement of key metabolites and mediators of microbial colonization. Electrochemical enzyme-based biosensors and microbead-based microfluidic assays expand the ability of organ-on-chip systems to perform detailed monitoring of metabolic parameters, including the formation of glutamine and glutamate (73, 74), the release of reactive oxygen species (75), and the secretion of various cytokines (76, 77). The determination of transepithelial electrical resistance (TEER) is another widely used method to assess barrier function and has been adapted in recent years for organ-on-chip platforms. In particular, the combination of impedance spectroscopy and electrical stimulation has been used to quantify cell layer capacitance as a measure of villi differentiation in gut-on-chip models, which could be applied across different gut-on-chip platforms (78).

**Figure 2:**
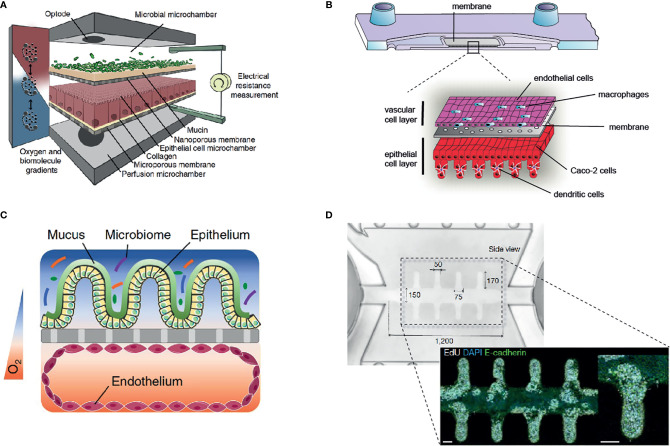
Overview of selected gut-on-chip models to study host-microbiota interaction *in vitro*. **(A)** The HuMiX gut-on-chip model allows the co-culture of anaerobic bacteria with an epithelial cell layer (68) (Creative Commons CC BY license). **(B)** Immunocompetent, multilayered gut-on-chip model comprising endothelial cells, epithelial cells, macrophages, and dendritic cells (12) (Copyright ^©^ 2019 Elsevier Ltd. All rights reserved). **(C)** Gut-on-chip model with oxygen tension gradient for the culture of a complex microbiome up to three days (11) (Copyright ^©^ 2019, The Author(s), under exclusive license to Springer Nature Limited). **(D)** Mini-intestine formed by organoid-derived epithelial cells, guided in growth by a perfused 3D hydrogel scaffold (37) (Copyright ^©^ 2020, The Author(s), under exclusive license to Springer Nature Limited).

**Figure 3:**
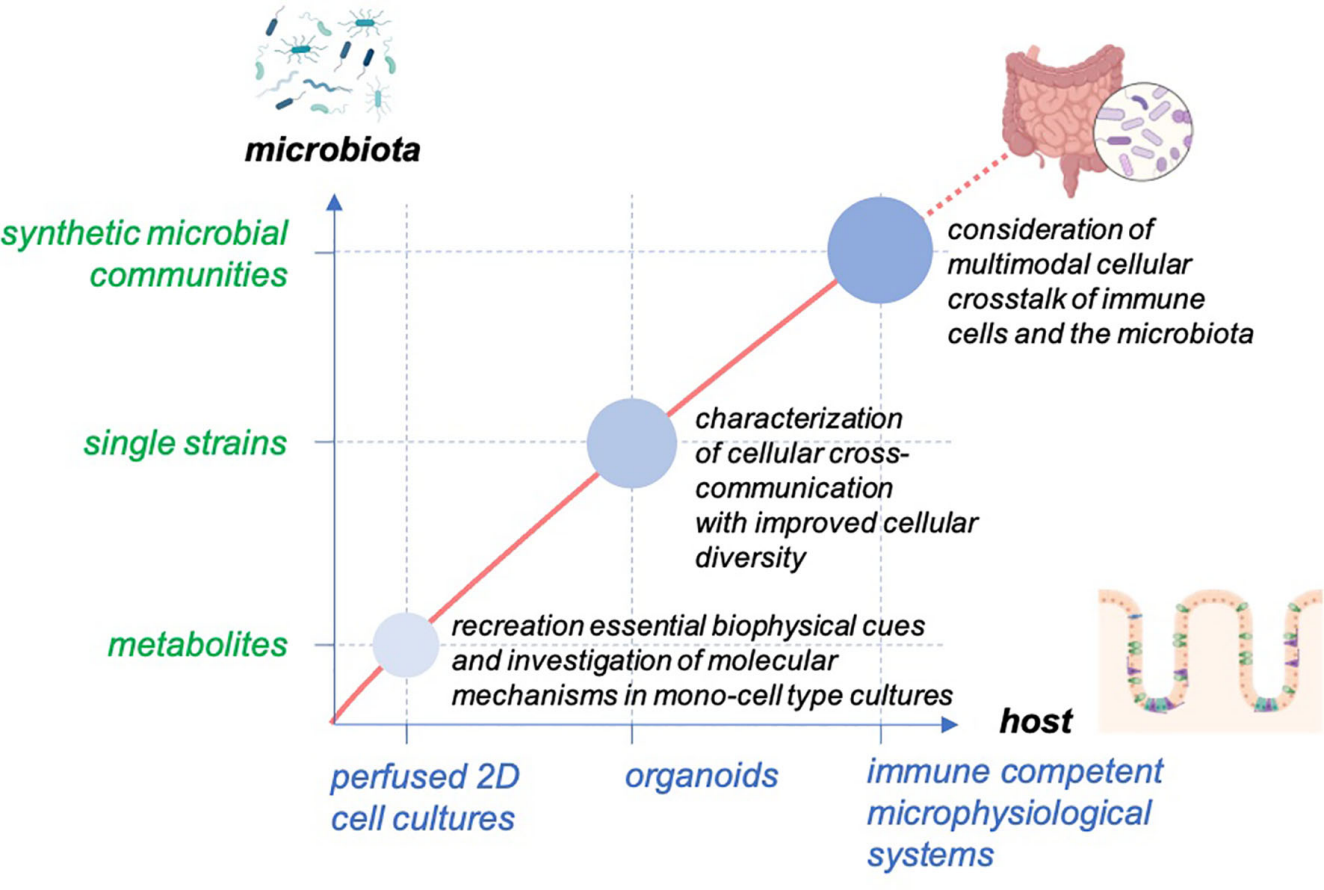
Concept for studying host-microbiome interaction in gut-on-chip. The models enable the balancing of the cellular complexity of host tissue and provide the technical basis to facilitate the outgrowth of host cells to self-organized organoid structures with high cellular diversity. The systems allow the scaling of the biological complexity of the host and the microbiota to study the interaction of the host tissue with the microbiota from microbial metabolites to single microbial strains and diverse microbial communities.

Gut-on-chip models have been used in recent years to better understand the complex interactions between the microbiota and their host. These systems allow balancing between robust and predictable approaches and the recreation of physiological-relevant culture conditions (**Figure 2**). The incremental nature of these models makes it relatively simple for them to be designed as “simple as possible and complex as required,” and they, therefore, offer additional flexibility in identifying contributing factors of host-microbiota interaction. The controlled escalation of biological complexity on the host side as well as in the composition of microbiota-derived factors and live microorganism communities will enable the elaboration and the proof of a complex interaction mechanism in a well-controlled and standardized environment provided by organ-on-chip platforms (**Figure 3**).

Examples of a successful application of this approach have already been provided. Sontheimer-Phelps et al. recently reported a system lined with primary patient-derived colonic epithelial cells (14). A mucus bilayer is built up in the chip by perfused epithelial cells and provides a suitable substrate for colonization with living microbiota to study host-microbiome interactions in the colon in more detail. Similarly, Shin et al. generated an anoxic-oxic interface on a chip inhabited with CaCo 2-cells and implemented *Bifidobacterium adolescentis* (79). The system is able to co-cultivate bacteria for up to one week without the impairment of epithelial performance. This represents an interesting platform to assess mechanistic questions regarding Bifidobacteria-host interactions in the neonate period in more detail. Similarly, Zhang et al. reported the co-culture of the obligate anaerobic bacterium *Faecalibacterium prausnitzii* with colon organoid-derived epithelial cells in their physiomimetic platform for up to four days. In the study, the authors were able to uncover the mechanisms of an anti-inflammatory host response related to butyrate release with the downregulation of the gene expression of histone deacetylase complexes, Toll-like receptors, and nuclear factor kB (NF-kB) (80). Different approaches have also been used to reconstitute the microbiome and its function *in vitro* in a more complex and detailed manner. A top-down approach consists of using the full microbiome derived from human stool samples, which would, in principle mimic the *in vivo* situation ideally. However, it remains challenging to characterize its full composition in a short period of time before transfer into an *in vitro* model, even under hypoxic conditions. Jalili-Firoozinezhad and co-workers managed to implement complex microbiomes in their *in vivo*-like system but pointed out that overgrowth and species shift due to artificial culture conditions might be problematic. Thus, a sophisticated pre-culture step (i.e., in large SHIME bioreactors) is still required to expand a defined microbiota prior to colonization experiments (11). However, these studies illustrate nicely the potential of advanced *in vitro* models to dissect the individual microbiota in humans.

Although conventional organoid cultures already provide invaluable insight into detailed ISC dynamics, their application for long-term studies or the dissection of host-microbiota interaction has some limitations. The long-term exposure of microbial metabolites or co-culturing living microorganisms in an enclosed lumen of organoids under static conditions favors the rapid overgrowth of bacteria, eventually resulting in the rupture and cell death of the organoids (32, 81). The microfluidic perfusion of gut-on-chip offers the ability of the long-term exposure of the tissue to microbiota-associated metabolites under well-defined conditions. Perfusion media can be supplemented with defined single molecules of microbial metabolites up to filtrates of stool samples, single strain microorganisms, or defined microbial communities. To better recapitulate the *in vivo* situation, gut-on-chip platforms could be leveraged to mimic the conditions of different sides of the gut by use of adult stem cells derived from specific sections (i.e., intestine, colon, etc.) to generate side-specific tissue (**Table 2**). Colonization with oxygen-scavenging bacteria in combination with the continuous sensing and regulation of oxygen levels would allow the maintenance of physiological-relevant oxygenation levels and a co-culture with defined anaerobic and facultative-anaerobic bacteria under homeostatic conditions.

**Table 2:** Key references of selected gut-on-chip systems with important characteristics to recapitulate the gut environment.

Reference	Cell source	Model characteristics and key findings
Shah et al. (68)	Caco-2	homeostatic colonization of epithelial cell layer with anaerobic bacteria under hypoxic conditions
Maurer et al. (12)	Caco-2, primary immune cells, and HUVECs	recapitulation of bacteria-fungal interaction in immunocompetent intestinal model
Shin et al. (79)	Caco-2	establishment of a hypoxic interface at an epithelial cell layer enabling co-culture with anaerobic bacteria (*Bifidobacterium adolescentis* and *Eubacterium hallii*)
Naumovska et al. (39)	Caco-2, human iPSC derived epithelial cells, human colon organoids	plate-based pumpless monolayer cell culture system allows membrane-free culture of intestinal epithelial cells
Wang et al. (34)	human small intestine organoid	recapitulation of the intestinal and colonic crypt region with the stem cell niche forming transit-amplifying epithelial cells within a confining hydrogel matrix
Wang et al. (35)	human colon organoids	
Sontheimer-Phelps et al. (14)	human colon organoids	culture of colon epithelial cells with differentiation of MUC2+ goblet cells and formation of colonic mucus bilayer under perfusion and cyclic strain
Kasendra et al. (9)	human duodenal organoids and human intestinal microvascular endothelial cells	analysis of gene expression profiles reveals closer recapitulation of *in vivo* conditions in tissue-on-chip compared to static organoid culture, endothelial cells support the formation of epithelial monolayers on-chip under perfusion and cyclic strain conditions
Nikolaev et al. (37)	murine proximal small intestinal organoids	hydrogel-confined crypt structures enable prolonged lifespan of organoids, improved cellular diversity under flow conditions with the development of rare cell types (microfold cells (M cells), immune-modulatory enterocytes, enteroendocrine cells)
Workman et al. (38)	hiPSC-derived human intestinal epithelial cells	formation of 3D crypt- and villus-like structures with self-patterned Paneth cells, goblet cells, enterocytes, enteroendocrine cells, transit-amplifying cells, and Lgr5+ cells under flow conditions, responsiveness to inflammatory triggers
Jalili-Firoozinezhad et al. (11)	Caco-2, human intestinal endothelial cells, human ileal organoids	tunable oxygen gradients allow the culture of obligate anaerobic bacteria (*Bacteroides fragilis*) and bacterial communities (human gut microbiota)
Zhang et al. (80)	human colon organoids	oxygen gradient allows stable co-culture of *Faecalibacterium prausnitzii* and identification of short-chain fatty acid butyrate mediated anti-inflammatory effects

## Modeling Human Intestinal Disease on-Chip

### Inflammatory Diseases

Gut-on-chip models offer a new avenue through which to study the pathophysiological mechanisms of gastrointestinal diseases. For example, patients suffering from Celiac disease (CeD), an autoimmune disease that affects about 1% of the human population, develop a strong inflammatory response causing severe damage to the small intestine upon the uptake of a gluten-containing diet (82). Still, the mechanistic details of the disease await further investigation before effective drugs can be developed. Moerkens et al. recently proposed an immunocompetent organ-on-chip model using both hiPSC and primary epithelial cells to mimic the genetic background and environmental factors more reliably. Using this system, it is now possible to determine the disease onset in CeD patients (83). Similarly, gut-on-chip models have been used to study the disease mechanism of inflammatory bowel disease (IBD), which includes Crohn’s disease (CD) and ulcerative colitis (UC), characterized by chronically relapsing intestinal inflammation and representing a worldwide healthcare problem with continually increasing incidence (84). A 3D gut model based on primary patient-derived colonic epithelial cells forming a mucus layer was used to recapitulate prostaglandin E2-dependent mucus volume expansion *via* the activation of the Na-K-Cl cotransporter 1 ion channel (14). In a similar study, the pathophysiology of DSS-induced inflammation was also recapitulated in the presence of *E. coli* (16). DSS cessation quickly induced the recovery of barrier integrity, villus formation, and mucus production, indicating that this model might also be suitable to study processes of mucosal regeneration in IBD. The effects of microbial colonization were also investigated with a mixture of commensal *Streptococcus thermophilus*, Bifidobacteria, and Lactobacilli (VSL#3) as well as pathogenic enteroinvasive *E. coli* (EIEC). In this study, the authors were able to replicate inflammation-associated tissue damage and the invasion of EIEC. Furthermore, they proved the growth-limiting effect of VSL#3 on EIEC colonization *in vitro* (85). Recently, our group has been able to further replicate the protective characteristics of *Lactobacillus rhamnosus* against the tissue infiltration of the opportunistic yeast *Candida albicans* in an immunocompetent gut-on-chip model. In addition to peripheral immune cells, this model also contained functional mucosal macrophages and dendritic cells integrated into the epithelial tissue layer (12). In contrast to conventional organoid cultures, chip-based ISC cultures enable long-term studies and perfusion with peripheral immune cells with superior accessibility and the continuous monitoring of key environmental factors to maintain and manipulate disease-related growth conditions. In combination with a homeostatic bacterial co-culture, these systems might serve in future studies as patient-specific tools to develop new therapeutics or probiotics that modulate the mucus barrier.

### Infectious Diseases

Gut-on-chip has further been used to study infectious diseases, including *Clostridioides difficile* infection (CDI), Enterohemorrhagic *E. coli* (EHEC), and viral infections. Infection with *Clostridioides difficile* (formerly *Clostridium difficile*) is the most common cause of hospital-acquired gastrointestinal infections (86, 87). While several animal models have been used to study CDI *in vivo*, the manipulation of infection processes and the direct assessment of host-bacterial interaction is challenging (88–90). Advanced gut-on-chip models have been described to investigate CDI. This model allows for the co-culture of primary epithelial and endothelial cells, with the addition of a complex microbiome under anaerobic culture conditions for several days (11). This is a promising translational tool to study the CDI infection process and associated damage in humans. As with CDI, infections with EHEC are frequent, with up 100,000 cases per year in the U.S. and are associated with several symptoms such as severe bloody diarrhea, hemorrhagic colitis, and hemolytic uremic syndrome (HUS). Interestingly, the microbiota seems to be a critical determinant of susceptibility to EHEC infection. Studies in mice revealed that not only is a 100,000-fold higher dose of the bacterium required (10^7^ microbes) but that the whole murine microbiome also needs to be depleted to induce symptoms. This pathology is quite different in humans, where as little as 10^2^ microbes are sufficient to cause severe symptoms (91–93). Tovaglieri et al. were able to recapitulate this phenomenon *in vitro* in a gut-on-chip model by co-culturing EHEC in the context of a murine or a human microbiome. The species-specific tissue damage induced by the human microbiome could be verified. This model was also used to identify several metabolites related to or even causative of the high susceptibility of humans to EHEC (13). Finally, human enteroviruses are responsible for an estimated 10–15 million infections and at least 30,000–50,000 hospitalizations per year (94). Viruses can replicate in the epithelial cells of the gastrointestinal tract and subsequently disseminate to secondary sites of infection, such as the respiratory tract (95). So far, no approved drugs for the treatment of enterovirus infections in humans besides polio exist (95). Villenave et al. used coxsackievirus B1 (CVB1) as a prototype enterovirus strain and established a human infection model based on a human gut-on-a-chip microfluidic device to study replication and infectious virus production *in vitro* (94). With this model, they were able to follow the infection of epithelial cells, the release of inflammatory cytokines, and the secretion of infectious virions. The model also permitted to reproduce the different susceptibility of epithelial and endothelial cell layers for viral infection by using separate microchannels, thus independently perfusing the vascular and epithelial sides of the polarized gut tissue. Gut-on-chip models have further just recently been used to study infection with human parasites. “Mini-intestines” fully accessible from the luminal side enabled colonization with Cryptosporidium parvum, an obligate parasite causing life-threatening diarrhea in immunocompromised adult hosts and in infants. The tissue model supported the full life cycle of the parasite, thereby allowing for the first time long-term studies in a primary cell-derived *in vitro* culture system (37).

With the emergence of the SARS-CoV2 virus and the COVID-19 pandemic, the need for sophisticated models to study viral infection in human tissue has become even more urgent. Human small intestinal organoids have proven to be valuable tissue models to study virus docking to angiotensin converting enzyme II and its replication within enterocytes. Combining ISC-derived organoids with the advantages of organ-on-chip would offer additional options to manipulate the viral infection route and provide different parameters to faithfully reflect the intestinal microenvironment, prolong the culture of organoids with the improvement of cellular differentiation, and offer a variety of additional monitoring and readout techniques available for gut-on-chip systems (96).

### Colorectal Cancer

The effects of short-chain fatty acids (SCFAs) released by probiotic *Lactobacillus rhamnosus GG* (LGG) bacteria on the growth of colorectal cancer cells was recently investigated in a version of the HuMiX model, a modular, microfluidics-based gut-on-chip model, which allows the co-culture of human and microbial cells and facilitates anaerobic culture conditions (97). Symbiotic treatment with a high-fiber diet was simulated in this model, and the generation of organics and SCFAs was assessed. Strikingly, it was demonstrated that SCFAs and lactate production was altered by a simulated high-fiber diet compared to a reference diet medium containing only simple sugars. The simulated high-fiber diet caused an increased expression of oncogenes and proinflammatory signaling in the absence of LGG bacteria, whereas, in the presence of LGG, both gene clusters were found to be significantly downregulated and associated with a reduced cell proliferation rate of primary colon-rectal cancer cells. This study illustrates the capability of gut-on-chip systems to precisely dissect the individual aspects of the microbiota-host crosstalk at the metabolic level. Hopefully, similar studies will further expand our insight into this complex relationship by identifying individual metabolic targets with potential as tailored pre- and probiotic-based therapeutic options. Uncovering individual beneficial metabolic profiles by applying hiPSC-based organ models and individualized microbiota surrogates in personalized *in vitro* models would represent a major step forward in the treatment of IBD and IBD sequelae such as colorectal cancer.

## Conclusions and Outlook

The intestinal tissue shows considerably variance in its architecture, cellular and molecular composition along the cephalocaudal axis, thereby creating various defined environmental niches populated with a specifically adapted microbiota (54, 60, 98). These niches are defined by differences in biophysical cues such as luminal oxygen levels or changes in pH and formed by adapted epithelial cells. Future gut-on-chip systems should be able to recapitulate this topography and allow the direct monitoring of environmental changes i.e., induced by changes in the microbiota composition and its impact on drug metabolization. It has been demonstrated that chip-based cultivation of organoid-derived cells could accelerate the formation of a self-organized epithelial microarchitecture, allowing the long-term culture with a living microbiota (11, 37, 79). These models have already proven to produce data similar to *in vivo* contexts in studies ranging from infectious disease modeling to drug metabolism (12, 13, 36, 41, 42, 94). The benefits and wide range of applications of the gut-on-chip models can be attributed to their versatility and their capability to increment parameters of various types, from multiple cell types to medium gradients, flow mechanics, or global topography. Despite these numerous advances in the complexification of microfluidics systems, many requirements still need to be met in order to obtain models with an *in vitro* microenvironment that closely mimics its *in vivo* counterpart.

Gut-on-chip technology offers unique options by providing the technical basis for a reliable and guided differentiation of stem cells to human intestinal tissue, combining the strengths of self-patterned stem-cell-based organoids with the ability to precisely regulated biochemical and biophysical cues in a spatiotemporal manner. The manipulation of the microenvironment by tissue engineering and the guided growth of stem cells derived from patients will create a new angle for the dissection of personalized host-microbiota interaction with scalable levels of complexity for the host and the microbiota. Optimizing fully defined and tunable hydrogel matrices as an alternative to the widely used Matrigel will allow the generation of organoids in a more standardized and reproducible way as a reliable cell source for gut-on-chip systems. Promising approaches have already demonstrated the potential of novel synthetic polymer matrices based on alginate (99), thrombin cross-linked fibrin gels (100) or PEG macromers decorated with maleimide groups (101).

The combination of advanced cell substrates and the precise control of differentiation conditions offered by microfluidic perfusion has great potential for improving stem cell differentiation and maturation to faithfully mimic epithelial cell layers (37). With the ability to monitor and perturb the milieu by manipulating isolated biophysical and biochemical cues in real-time these systems provide a deeper mechanistic insight into stem cell differentiation and effects of changes in the microenvironment on tissue formation its interaction with the microbiota. Further, gut-on-chip will help identify the molecular and cellular targets of disease-related mechanisms for individual patients that cannot be resolved in similar detail in animal models. Improvements of the materials used for chip manufacturing will also increase its relevance for drug testing. Polydimethylsiloxane so far widely used for organ-on-chip is known to non-specifically bind small hydrophobic molecules, which potentially causes bias in drug-absorption and metabolization studies (102, 103). Advanced microfabrication processes using i.e., thermoplastic polymers with more inert properties, will foster the acceptance of gut-on-chip models for drug screening (104).

Clearly, organ-on-chip and in particular complex gut-on-chip models have several limitations. In contrast to conventional organoid cultures, their usability in high-throughput testing is limited.

The ORCHID consortium thus elaborated recommendations for the development and use of future organ-on-chip models as part of a European Roadmap (105). End users need to be provided with customizable platforms “fit-for-purpose” and provided with tailored training programs. ISC-based gut-on-chip as well as ISC-derived organoids represent reductionistic models, which do not reflect the full physiology offered by animal models. Currently available systems thus need to be selected based on the scientific context of the study i.e., the pathogen that is studied or its targeted cell type. The use of standardized and open platform technology will benefit in the future by the establishment of independent testing centers for the qualification and characterization of organ-on-chip models (105).

In the case of organ-on-chip modeling of the gastrointestinal tract, a more “complete” model would need to be composed of adjacent channels with human microvascular endothelium, tissue-resident immune cells, commensal or pathogenic bacteria, and the application of cyclic mechanical forces that mimic peristalsis-like deformations. The additional presence of muscle or nervous system cells could also contribute to create such a faithful model. To this date, there is no system available which is capable to recapitulate every aspect of the gut, considering biophysical, biochemical, immunological and microbiota derived cues. However, the combination of stem cell-based organoid culture and organ-on-chip will help pave the way to significantly expand our knowledge in the development and maintenance of intestinal tissue and its role in the onset of related diseases. Several challenges, such as the uniformization of the systems and its standardization, need to be overcome to allow researchers and non-organ-on-chip specialists easy access to this technology and to allow its routine use in biomedical research.

## Author Contributions

FS, EL, MK, HK, MM, and AM contributed to the writing of the manuscript. MM and AM conceptualized, supervised, and edited the manuscript. All authors contributed to the article and approved the submitted version.

## Funding

This work was financially supported by the Deutsche Forschungsgemeinschaft through the Cluster of Excellence “Balance of the Microverse” under Germany’s Excellence Strategy - EXC 2051 - Project-ID 690 390713860 and the European Commission through the Marie Skłodowska-Curie Actions (MSCA) Innovative Training Network EUROoC (Grant no. 812954) to AM. This work was also supported by the Agence Nationale de la Recherche ANR-17-CE14-0021 (SyNEDI to MM) and a “New Team” grant (BOGUS to MM) from the Bioregate Regenerative Medicine Cluster, University of Nantes and Région Pays de la Loire.

## Conflict of Interest

The authors declare that the research was conducted in the absence of any commercial or financial relationships that could be construed as a potential conflict of interest.

## Publisher’s Note

All claims expressed in this article are solely those of the authors and do not necessarily represent those of their affiliated organizations, or those of the publisher, the editors and the reviewers. Any product that may be evaluated in this article, or claim that may be made by its manufacturer, is not guaranteed or endorsed by the publisher.
